# Urine human papillomavirus testing for cervical screening in a UK general screening population: a diagnostic test accuracy study

**DOI:** 10.3399/BJGP.2025.0105

**Published:** 2025-11-18

**Authors:** Jennifer C Davies, Suzanne Carter, Jiexin Cao, Maya Whittaker, Helena O’Flynn, Clare Gilham, Peter Sasieni, Alexandra Sargent, Emma J Crosbie

**Affiliations:** 1 Gynaecological Oncology Research Group, Division of Cancer Sciences, Faculty of Biology, Medicine and Health, University of Manchester, Manchester, UK; 2 Department of Obstetrics and Gynaecology, St Mary's Hospital, Manchester University NHS Foundation Trust, Manchester Academic Health Science Centre, Manchester, UK; 3 Bowland Medical Practice, Manchester, UK; 4 Faculty of Epidemiology and Population Health, London School of Hygiene and Tropical Medicine, London, UK; 5 Centre for Cancer Screening, Prevention and Early Diagnosis, Wolfson Institute of Population Health, Queen Mary University of London, London, UK; 6 Cervical Screening Laboratory, Clinical Sciences Centre, Manchester University NHS Foundation Trust, Manchester Academic Health Science Centre, Manchester, UK

**Keywords:** cervical cancer, diagnostic test accuracy, human papillomavirus testing, screening, self-sampling, urine

## Abstract

**Background:**

Cervical screening uptake is decreasing in the UK, with only 67.5% of those eligible under 50 years old attending in 2022. Barriers include restricted access to screening appointments and poor acceptability of the speculum examination. Urine self-sampling is an alternative cervical screening method that has the potential to improve uptake.

**Aim:**

To determine the clinical performance and acceptability of human papillomavirus (HPV)-tested urine for cervical screening in a UK general screening population.

**Design and setting:**

Prospective, cross-sectional diagnostic test accuracy study in North-West England.

**Method:**

Urine was self-collected using a first-void urine (FVU) collection device (DNA Genotek Colli-Pee® 10 ml with urine conservation medium) before obtaining matched routine cervical screening samples. HPV testing used Roche Cobas® 8800 at cervical sample thresholds. A questionnaire evaluated urine self-sampling acceptability. HPV-positive cervical samples underwent reflex cytology, managed under standard NHS protocols, and clinical outcomes were collected.

**Results:**

In total, 1517 participants provided matched urine and cervical samples. There were 207 of 1517 (13.6%) cervical and 245 of 1517 (16.2%) urine samples that were HPV positive with a 1.6% (*n* = 25/1517) incidence of cervical intraepithelial neoplasia (CIN)2+ following colposcopic assessment (*n* = 80). The specificity of urine was non-inferior (*P* = 0.0004) to the specificity of cervical samples at 85.19% (95% confidence interval [CI] = 83.28 to 86.95) versus 87.80% (95% CI = 86.03 to 89.42), giving a relative specificity of 0.97 (95% CI = 0.95 to 0.99). Urine detected 24 of 25 (96.0%) participants with CIN2+. In the future, 41.6% (*n* = 575/1382) of participants would prefer current cervical screening, compared with 30.0% (*n* = 414/1382) with no preference and 28.4% (*n* = 393/1382) preferring urine self-sampling.

**Conclusion:**

HPV-tested urine showed non-inferior specificity to cervical samples in a general screening population. Urine self-sampling was acceptable to current attenders but some prefer traditional screening, making choice an important consideration for policymakers.

## How this fits in

The switch from primary cytology to primary human papillomavirus testing has enabled innovations in self-sampling for cervical screening. This study shows that urine self-collected with a first-void urine collection device has similar diagnostic test accuracy and acceptability to cervical sampling in a general screening population. Urine self-sampling has real-world potential as an alternative cervical screening option.

## Introduction

Cervical screening has successfully reduced cancer-specific mortality.^1^ However, UK uptake is currently 67.5% and declining.^2,3^ Barriers include restricted access to screening appointments^3^ and poor acceptability of the speculum examination.^4^ Primary human papillomavirus (HPV) testing has enabled the development of self-sampling methods for cervical screening. Urine self-sampling shows promise as an alternative cervical screening method that may reduce barriers and improve uptake.^5^

Research to date has mainly focused on optimising urine collection and processing for HPV testing.^6–12^ First-void urine (FVU) device-collected urine is superior to standard pot-collected urine for cervical intraepithelial neoplasia (CIN)2+ detection,^13^ likely secondary to more reliable collection of peri-urethral mucus containing HPV-infected cellular debris.^14^ A systematic review pooling 11 159 women reported a relative sensitivity of 0.84 (95% confidence interval [CI] = 0.78 to 0.91) and relative specificity of 1.06 (95% CI = 1.03 to 1.10) for HPV detection in urine versus cervical samples, significantly improved with the use of a FVU collection device to 0.98 (95% CI = 0.91 to 1.05) and 1.03 (95% CI = 0.90 to 1.19), respectively.^15^ Nearly all studies to date have been conducted in high-risk colposcopy populations. The aim of this study was to determine the clinical performance and acceptability of urine self-sampling for cervical screening in a UK general screening population.

## Method

### Trial design and participants

ACES Primary Care was a prospective, cross-sectional diagnostic test accuracy study carried out in a general cervical screening population in North-West England between March 2022 and April 2024.

Potential participants were identified from clinic lists in eight primary care facilities and three hospital clinics across North-West England. Eligible individuals were aged 24–70 years and attending routine cervical screening. The study included people attending for annual recall (previously HPV positive, cytology normal) but excluded test-of-cure patients. Patient and public involvement and engagement informed study concept, design, and participant-facing materials. All participants provided written, informed consent to take part.

### Clinical procedures

Participants provided matched urine and cervical samples at the same visit. Urine samples were collected first. Participants were asked to collect their urine sample at least 1 h after their last void. They were shown how to use an FVU collection device before self-collecting their sample in the clinic bathroom. The FVU collection device was a 10 ml Colli-Pee® (DNA Genotek, Canada) containing urine conservation medium to immediately stabilise the sample pending laboratory transfer and testing. Matched healthcare practitioner-collected cervical liquid-based cytology samples were obtained at speculum examination using a Rovers Cervex-Brush® (Oss, Netherlands) and suspended in 20 ml of ThinPrep® solution (PreservCyt Solution, Hologic, UK) as per routine cervical screening.

After urine and speculum-based cervical sampling had been performed, participants were asked to complete a paper questionnaire during the visit. The four-page questionnaire (see Supplementary Information S1) comprised five sections that included some questions on a five-point Likert scale: knowledge about HPV, acceptability of cervical and urine sampling, future preference for sampling method, cervical screening in the past, and about you. Qualitative data were not collected.

### Laboratory analyses

Urine and cervical samples were transferred to Manchester University NHS Foundation Trust Cervical Screening Laboratory and stored at room temperature before being tested for high-risk HPV on average 5.9 days (range 0–28) after collection. After inverting the urine sample several times, a 2 ml aliquot was transferred to secondary tubes compatible with the Roche Cobas® 8800 testing platform. Cervical samples were tested according to the manufacturer’s instructions and urine samples were tested off-label using the manufacturer’s preset cervical sample cycle thresholds for HPV 16, HPV 18, HPV Other (HPV 31, 33, 35, 39, 45, 51, 52, 56, 58, 59, 66, and 68), and β-globin DNA. Lower cycle threshold values correlate with a higher sample viral load. Initial analyses included all urine samples.

### Clinical outcomes

Only cervical sample results were returned to the participant and used for clinical management according to the NHS Cervical Screening Programme (CSP). HPV-negative results required no further action and participants were returned to routine recall. HPV-positive cervical samples were triaged by cytology as per the NHS CSP protocol. If cytology was abnormal or inadequate, diagnosis was determined by colposcopic findings and/or histology, where applicable. If cytology was normal, a diagnosis of HPV infection was recorded unless this was their third consecutive HPV positive/normal cytology screen, in which case they were referred to colposcopy. All other individuals with HPV positive/cytology normal results will be invited for repeat HPV tests after 12 months as per the NHS CSP.

### Statistical analysis

The primary outcome was the diagnostic test accuracy of urine HPV testing for cervical screening within a general screening population, in particular, specificity, relative specificity, and sensitivity for CIN2+ detection, a clinically important outcome. Secondary outcomes included urine and cervical HPV test concordance and acceptability of urine self-sampling in current cervical screening attendees. A sample size calculation determined that a study of 1500 cervical screening attendees would have 91% power to show a relative specificity of ≥96.5% compared with matched cervical samples for CIN2+ detection, assuming that the true specificity is 98%. This study size is sufficient according to the 800 suggested by Meijer *et al* ^16^ and the 1000 suggested by HPValidate.^17^ To allow direct comparison with the HPValidate study, 90% CIs were also calculated that assessed non-inferiority at the 1-sided *α* = 0.05 level.

Absolute and relative clinical test accuracy were calculated with accompanying 95% CIs. A non-inferiority score test was used to assess test accuracy between matched FVU and cervical samples. The margin of non-inferiority for relative specificity was set to 0.95 (as per the threshold suggested by HPValidate^17^) so that under the null hypothesis non-inferiority was defined as relative specificity ≤0.95. The study was not powered to test for non-inferiority of sensitivity.^16,18^ Concordance of HPV positivity between urine and cervical samples was determined using Cohen’s Kappa (κ) statistical test and categorised as follows: *κ* ≤0.20, poor; 0.21 ≤*κ* ≤0.40, fair; 0.41 ≤*κ* ≤0.60, moderate; 0.61 ≤*κ* ≤0.80, good; and *κ* ≥0.81, excellent. McNemar’s test was used to compare paired proportions and χ^2^ test was used otherwise. Statistical analyses were performed with Stata (version 18) and GraphPad Prism (version 10.3.1).

## Results

### Participant demographics

**Figure 1.**
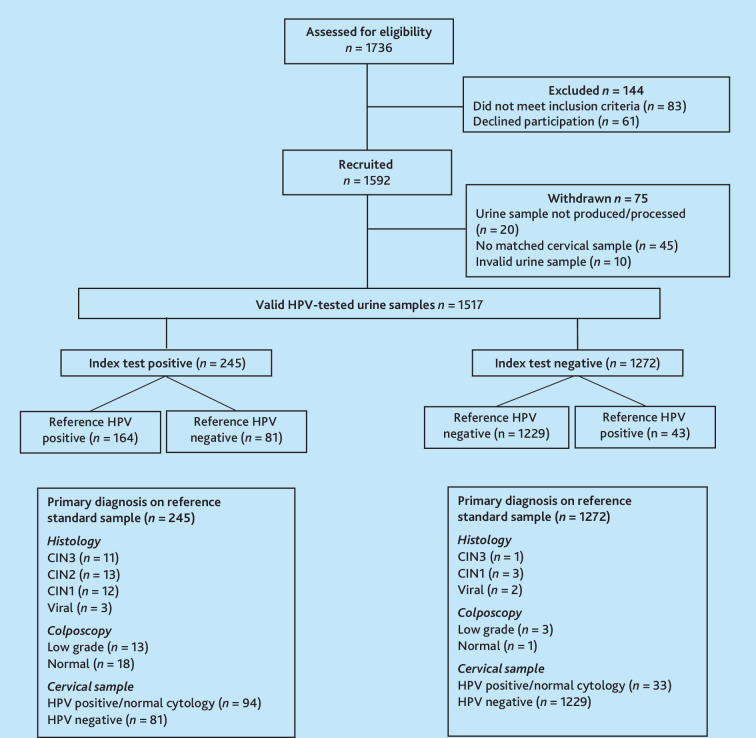
Study flowchart of participants and samples. CIN = cervical intraepithelial neoplasia. HPV = human papillomavirus.

Between March 2022 and April 2024, 1736 people were assessed for eligibility and 1592 recruited into the study (Figure 1). Participants were withdrawn if either a urine (*n* = 20) or cervical sample (*n* = 45) were not available for analysis. In total, 1517 matched urine and cervical samples were included in the final analysis, after additional exclusions based on an invalid urine result (*n* = 10).

The final study population had a median age of 37 years (interquartile range 30–45 years). Self-reported ethnicity of the participants was White (*n* = 1057/1517; 69.7%), Asian (*n* = 139/1517; 9.2%), Black (*n* = 97/1517; 6.4%), mixed (*n* = 72/1517; 4.7%), Arab (*n* = 12/1517; 0.8%), other ethnic identities (*n* = 8/1517; 0.5%), or not recorded (*n* = 132/1517; 8.7%). Most participants were employed (*n* = 1126/1386; 81.2%), heterosexual (*n* = 1239/1358; 91.2%), and identified as women (*n* = 1379/1390; 99.2%); 10 of 1517 (0.7%) were non-binary or transgender. In total, 524 of 1507 (34.8%) participants self-reported as smokers or ex-smokers and 384 of 1517 (25.3%) were HPV vaccinated, 85.9% (*n* = 330/384) of whom before they were 17 years of age.

### Concordance for HPV in urine and cervical samples

Most cervical samples were HPV negative (*n* = 1310/1517; 86.4%); HPV-positive samples (*n* = 207/1517; 13.6%) were more common in younger people (see Supplementary Table S1). Reflex cytology on the 207 HPV-positive cervical samples showed 4.8% (*n* = 10) high grade, 21.3% (*n* = 44) low grade, 5.8% (*n* = 12) borderline, 4.3% (*n* = 9) inadequate, and 63.8% (*n* = 132) normal.

The mean cycle threshold values (see Supplementary Figure S1) were higher in urine than cervical samples, resulting in positive differences in mean cycle threshold values of 2.39 for β-globin (*P*<0.001), 1.86 for HPV 16 (*P* = 0.9), 1.56 for HPV 18 (*P* = 0.7), and 2.20 for HPV Other (*P*<0.0001) between sample types.

Supplementary Tables S2 and S3 show good HPV concordance between FVU device-collected urine and matched cervical samples (91.8% overall agreement, *κ* = 0.68). However, urine samples were more likely to be HPV positive than cervical samples (*n* = 245/1517, 16.2% versus *n* = 207/1517, 13.6%; McNemar *P* = 0.0006) (Table 1). Most samples were positive for non-HPV 16/18 genotypes only (74.5% of positive urine samples). There were no cases of HPV 16 or HPV 18 among vaccinated participants. A higher proportion of non-HPV 16/18 infections were observed in younger (aged <35 years) unvaccinated participants than older (aged ≥35 years) unvaccinated participants (*n* = 55/61, 90.2% versus *n* = 57/83, 68.7%, respectively; *P* = 0.002). The prevalence of non-HPV 16/18 infections was similar in the self-reported vaccinated (*n* = 63/339, 18.6%) and non-vaccinated (*n* = 55/318, 17.3%) participants aged <35 years (*P* = 0.7). A similar prevalence of HPV 16 and HPV 18 was seen in unvaccinated younger (3.1% [*n* = 10/318] for HPV 16 and 0.9% [*n* = 3/318] for HPV 18) and older (2.6% [*n* = 22/860] and 1.2% [*n* = 10/860], respectively) participants.

**Table 1.** High-risk human papillomavirus type by sampling method and self-reported vaccination status^a^

			Aged <35 years				Aged ≥35 years	
	**All participants (** ***N*** **= 1517)**		**HPV vaccinated** ^**b**^ **(** ***N*** **= 339)**		**Unvaccinated (** ***N*** **= 318)**		**Unvaccinated (** ***N*** **= 860)**	
**Characteristic**	***n***	**%**	***n***	**%**	***n***	**%**	***n***	**%**
**Cervical sample positive**								
HPV negative	1310	86.4	276	81.4	257	80.8	777	90.3
Any HPV	207	13.6	63	18.6	61	19.2	83	9.7
HPV 16	32	2.1	0	0.0	10	3.1	22	2.6
HPV 18	13	0.9	0	0.0	3	0.9	10	1.2
HPV other	175	11.5	63	18.6	55	17.3	57	6.6
**Urine sample positive**								
HPV negative	1272	83.8	272	80.2	251	78.9	749	87.1
Any HPV	245	16.2	67	19.8	67	21.1	111	12.9
HPV 16	44	2.9	0	0.0	11	3.5	33	3.8
HPV 18	23	1.5	0	0.0	7	2.2	16	1.9
HPV other	200	13.2	67	19.8	58	18.2	75	8.7

^a^Samples positive for multiple HPV types are counted more than once. ^b^Including six participants who were aged ≥35 years, but vaccinated aged 18–39 years. None tested HPV positive. HPV = human papillomavirus.

### Clinical performance of Roche Cobas® 8800 HPV testing

Clinical outcomes were determined by the cervical HPV/cytology test result (*n* = 1437/1517, 94.7%), colposcopic impression (*n* = 35, 2.3%), and histology (*n* = 45, 3.0%) (see Supplementary Table S1). In total 80 individuals underwent colposcopy (*n* = 61/66 following abnormal cytology, *n* = 16/17 with persistently HPV positive/normal cytology, and *n* = 3/3 following inadequate cytology) and 25 of 1517 (1.6%) cervical intraepithelial neoplasia-(CIN)2+ lesions were identified.

When applying the manufacturer’s cycle threshold for HPV positivity for cervical samples, urine had a specificity of 85.19% (95% CI = 83.28 to 86.95) and a relative specificity of 0.97 (95% CI = 0.95 to 0.99) compared with cervical samples, which had a specificity of 87.80% (95% CI = 86.03 to 89.42) for CIN2+ detection (Table 2). The score test showed evidence against inferiority set at the 0.95 threshold (*P*<0.01). The sensitivity for CIN2+ detection was 96.00% (95% CI = 79.65 to 99.90; *n* = 24/25) and for CIN3 was 91.67% (95% CI = 61.52 to 99.79; *n* = 11/12).

**Table 2.** Clinical sensitivity (CIN2+ and CIN3+) and specificity (<CIN2) of the Roche Cobas® 8800 HPV assay in cervical samples and FVU collected with the FVU collection device^a^

	CIN2+		CIN3+		<CIN2		
**Sample**	***n*** **/** ***N***	**Sensitivity, % (95% CI)**	***n*** **/** ***N***	**Sensitivity, % (95% CI )**	***n*** **/** ***N***	**Specificity, % (95% CI)**	**Relative specificity** ^**b**^ **(95% CI)** ***P*** **-value** ^**c**^
**Cervical**	25/25	100 (86.28 to 100)	12/12	100 (73.54 to 100)	1310/1492	87.80 (86.03 to 89.42)	—
**Urine**	24/25	96.00 (79.65 to 99.90)	11/12	91.67 (61.52 to 99.79)	1271/1492	85.19 (83.28 to 86.95)	0.97 (0.95 to 0.99) 0.0004

^a^Exact binomial 95% CI. ^b^Relative sensitivity and specificity are restricted to the groups of paired samples. ^c^McNemar’s paired *P-*value. CIN = cervical intraepithelial neoplasia. FVU = first-void urine. HPV = human papillomavirus.

### Acceptability of cervical screening methods

Survey responses from the 1517 participants with matched urine and cervical samples indicated that more participants found urine self-sampling easy (*n* = 1021, 67.3%) compared with routine cervical screening (*n* = 832, 54.8%). Fewer participants found urine self-sampling uncomfortable (*n* = 24, 1.6%) compared with routine cervical screening (*n* = 373, 24.6%). Fear of what the test might find (*n* = 301/1395, 21.6%), embarrassment (*n* = 333/1399, 23.8%), worry about pain or discomfort (*n* = 510/1403, 36.4%), difficulty making an appointment (*n* = 452/1397, 32.4%), and taking time off work (*n* = 476/1394, 34.1%) were the main barriers participants cited for delaying screening in the past. A previous bad experience (*n* = 87/1394, 6.2%) and being asymptomatic (*n* = 133/1391, 9.6%), were less commonly reported barriers.

**Figure 2.**
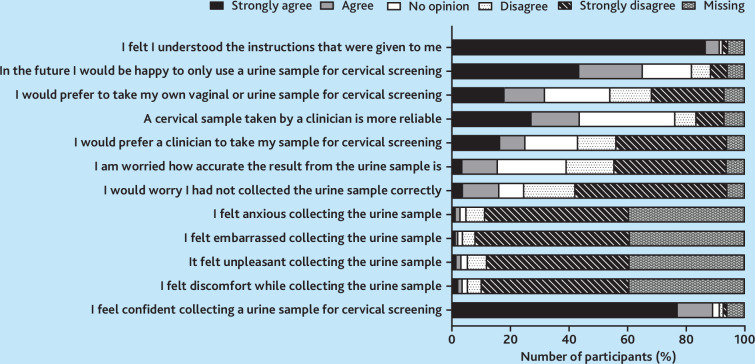
The acceptability of FVU device-collected urine for cervical screening. FVU = first-void urine.

In the future, 41.6% (*n* = 575/1382) of participants would prefer a healthcare professional to take their sample, compared with 30.0% (*n* = 414/1382) with no preference, and 28.4% (*n* = 393/1382) who would prefer to self-sample (Figure 2). Overall, 54.8% (*n* = 498/908) strongly or somewhat agreed that offering a choice of screening methods would improve their cervical screening experience.

## Discussion

### Summary

The ACES Primary Care study shows that self-sampled urine collected with an FVU collection device and tested for HPV using Roche Cobas® 8800 may have non-inferior diagnostic test accuracy compared with a matched cervical sample in a UK general screening population. Compared with the referent cervical sample, urine had a relative specificity of 97% and identified 24/25 CIN2+ lesions.

Urine was broadly acceptable to current attenders of cervical screening; however, 41.6% would prefer routine cervical screening, making choice important in any future cervical screening programme. Future studies should explore the potential of urine self-sampling to improve cervical screening uptake in current non-attenders and its potential impact on cervical screening programme effectiveness if offered as a choice to everyone.

### Strengths and limitations

To the authors’ knowledge, this is the first study evaluating HPV testing of optimised FVU device-collected urine in a general cervical screening population with a direct comparison with matched cervical samples and clinical outcomes. The 10 ml FVU collection device containing non-toxic preservative stabilises the sample at ambient temperature before laboratory testing and its compact design enables postal delivery through a standard UK letterbox for at-home sampling.

This study included 1517 same-day matched urine and cervical samples, minimising the risk of discordance because of HPV-infection status changing over time. HPV testing used the Roche Cobas® 8800 testing platform, which is one of the assays currently used by the NHS CSP, enabling direct clinical translatability. Samples were stored at ambient room temperature before batch testing with very few invalid results (<1%), showing promising stability and potential for at-home collection.

This is the largest study to date to evaluate urine self-sampling and provides important insight into the potential future sampling choices of current attenders of an urban general cervical screening population. The study population is representative of Manchester, UK, a diverse urban area with >30% of global majority ethnicities represented, bringing real-world relevance when considering future cervical screening policy change.

Limitations of this study relate to the recruitment of current attenders of cervical screening, precluding its ability to ascertain the acceptability of urine self-sampling within non-attenders including people with physical or intellectual disability and gender-diverse individuals, where it has most potential to improve outcomes. All urine samples were collected in clinic following verbal instruction; in this study the authors did not test home-based collection of urine self-samples and therefore cannot comment on the pragmatic safety and logistical issues that may arise with the transportation of wet samples. Only HPV-positive cervical samples triggered further investigation and therefore clinical test accuracy was positively skewed towards healthcare-collected cervical sampling. The assumption that urine HPV-positive/cervical HPV-negative samples (*n* = 81/1517, 5.3% of participants) were false positives may be inaccurate and long-term follow-up is needed to track clinical outcomes in these individuals.

Only one HPV assay was used in this study and, as several are in routine NHS use, further work is required to validate their analytical performance. The manufacturer’s cervical sample thresholds were used in this study to define HPV positivity in urine, which disadvantages urine as a sample type as HPV viral load is lower. Bespoke urine cycle threshold positivity thresholds may improve clinical test performance.

### Comparison with existing literature

The Alternative CErvical Screening (ACES) Colposcopy study, by the same author group, demonstrated that an FVU collection device is needed for optimal urine collection, significantly improving sensitivity for CIN2+ detection compared with the standard pot (90.3% versus 73.4%, *P* = 0.0005).^13^ The current study is the first study to evaluate the clinical test accuracy of HPV-tested FVU device-collected urine in a general cervical screening population. The only previous study, Papillomavirus Dumfries and Galloway (PaVDaG), collected urine in a standard pot and found an inferior diagnostic test accuracy compared with referent cervical samples.^19^

The ACES, VALHUDES, PREDICTORS 5.1, and EVAH studies demonstrate that FVU-collected urine and cervical samples have similar diagnostic test accuracy for CIN2+ detection in colposcopy referral populations across a range of HPV assays.^6,12,13,20–22^ Test sensitivity could be further improved with the application of bespoke urine polymerase chain reaction cycle thresholds as exemplified by the VALHUDES studies where Abbott RealTime^®^,^6^ BD Onclarity^®^,^12^ and Alinity^®^ m^20^ all achieved relative sensitivities of 1.00 compared with matched cervical samples. The Roche Cobas® assay did not allow the exploration of bespoke urine cycle threshold positivity thresholds to improve test accuracy. Other HPV assays approved for use in the NHS and other national cervical screening programmes now require validation for urine-based testing including those utilising mRNA-based technology.

The HPValidate study compared the performance of several vaginal self-sampling/HPV testing workflows for potential NHS adoption. It assessed performance using prespecified relative sensitivity non-inferiority lower 95% CI bound thresholds of 90% (versus clinician collected, HPV) and 75% (versus clinician collection, cytology), and 95% for relative specificity.^17^ Four testing workflows met the lower sensitivity thresholds and one met the higher sensitivity threshold, and these workflows are among those being considered by the National Screening Committee for offering HPV self-sampling to underscreened people in the NHS CSP.

With 1492 matched <CIN2 samples, and adopting 90% CIs instead of the more standardly used 95% to allow direct comparison with the workflows tested in HPValidate, this ACES Primary Care study estimated a non-inferior relative specificity (97.0%, 90% CI = 95.7 to 98.4) of urine HPV testing compared with cervical screening (*P* = 0.01) that was higher than both vaginal samplers tested with Cobas® in the HPValidate study.^17^ The authors of the current study previously reported a relative sensitivity of 91.8% based on 124 patients with CIN2+ in the ACES Colposcopy study.^13^ Taking these two ACES studies together, the relative sensitivity of urine HPV testing for CIN2+ is 92.5% (90% CI = 88.7 to 96.5), which is similar to the higher performing workflows tested in the HPValidate study,^17^ showing potential for Colli-Pee® collected urine tested for HPV with Roche Cobas® as a self-samplng workflow for cervical screening within the NHS and other healthcare systems.

Brentnall and colleagues argue that validation of self-sampling methods requires a flexible, staged, and adaptive design for evaluation.^23^ The ACES studies meet the second stage of evaluation, comparing HPV-tested FVU device-collected urine with healthcare-collected cervical samples according to clinical outcomes from HPV-positive cervical samples. This design returns a pessimistic relative test accuracy for urine and demonstrating non-inferior clinical performance is very promising for real-world clinical effectiveness.^24^

This is the first study in the UK to assess the concurrent acceptability of urine self-sampling for cervical screening within a primary care population. The data in the current study show that 41.6% (*n* = 575/1382) preferred routine cervical screening but 28.4% (*n* = 393/1382) of individuals preferred urine sampling, and 30.0% (*n* = 414/1382) had no preference. This is perhaps unsurprising given that the study participants were attending their routine cervical screening appointment when approached to take part. A recent English cross-sectional questionnaire study showed that current attenders were less likely to choose self-sampling for cervical screening than under- and never-screened individuals (41.0% versus 71.1% versus 70.1%, respectively), which is in keeping with the data in the current study.^25^ The range of preferences highlights the importance of choice of cervical sampling method for cervical screening participants, especially given that over half (54.8%, *n* = 498/908) of the participants in the current study strongly or somewhat agreed that offering a choice of screening methods would improve their cervical screening experience. The preference for choice is echoed in other UK-based studies of vaginal self-sampling within screening populations.^26,27^ How to offer choice requires exploration within behavioural science implementation studies and must be tailored to ensure that groups less likely to attend have special focus, to ensure narrowing rather than a widening of the screening inequity gap.

### Implications for research and practice

Home-based urine self-sampling has the potential to eliminate many barriers to cervical screening including access, inconvenience, and poor acceptability of the speculum examination.^28,29^ Urine self-sampling reduces the financial and environmental costs associated with cervical screening.^30,31^ It may also offer a viable option in resource-poor healthcare systems where the burden of cervical disease is greatest, potentially providing a point-of-care test to triage women for same-day investigation and treatment, thereby reducing loss to follow-up.

A positive urine self-sample is likely to require a routine cervical sample for reflex cytology and colposcopy triage. Studies have shown that up to 90% of research participants who test HPV positive through self-sampling subsequently attend for cervical sampling and colposcopy triage, including never- and underscreened individuals.^27,32^ Methylation testing is an emerging molecular triage tool that can distinguish low- from high-grade cervical lesions in urine samples.^33,34^ Further exploration and validation is required but, if methylation testing were found to be as effective or superior to cytology for triaging HPV-positive samples, it could replace the need for a speculum examination for most women found to be HPV positive at self-sampling. This would enable reflex testing of residual, stored urine samples, reducing cost and delays, and providing a one-stop fully automated, high-throughput molecular test for cervical screening using urine samples self-collected in the privacy of a person’s home.

In conclusion, this study has demonstrated that HPV-tested urine collected with an FVU collection device may have non-inferior diagnostic test accuracy to healthcare-collected cervical samples in a UK general cervical screening population. Urine sampling is broadly acceptable to current attenders of cervical screening; however, some still prefer speculum-based clinician cervical sampling, highlighting the importance of choice in future versions of the cervical screening programme. Research should now focus on measuring the impact of urine self-sampling among individuals who currently do not attend for cervical screening, where this novel intervention stands to make the biggest difference.^35^

## Data

Study data were collected and managed using Research Electronic Data Capture (REDCap) electronic data capture tools hosted at the University of Manchester. REDCap is a secure, web-based software platform designed to support data capture for research studies, providing 1) an intuitive interface for validated data capture; 2) audit trails for tracking data manipulation and export procedures; 3) automated export procedures for seamless data downloads to common statistical packages; and 4) procedures for data integration and interoperability with external sources. A fully anonymised dataset for this study will be made available in the research data repository, Figshare.
